# Discussion upon Alcohol in the Philadelphia County Medical Society

**Published:** 1881-09

**Authors:** 


### DISCUSSION UPON ALCOHOL IN THE PHILADELPHIA COUNTY MEDICAL SOCIETY.

Dr. George Hamilton regarded the nourishment of the patient as the main point in the treatment of exhausting diseases: stimulants might aid nutrition, but cannot take the place of food. In declining years of life a moderate amount of alcohol may be of service, but in youth it should be used with the greatest caution. The recommendation of Todd had been too closely followed, and he could now see signs of a reaction against indiscriminate stimulation. Typhoid fever is most likely to occur at from sixteen to twenty-one years of age, which also is a very fatal period. The mucous membrane of the alimentary tract being at this age unaccustomed to alcohol, the greatest caution should be observed in giving large amounts of stimulants. It is usually recommended to be given early in the disease, which he regarded as wrong. Even in phthisis it should be used with care. He believed the that the physician is often unjustly blamed for having recommended stimulants to persons who form the habit of drinking to excess; and he cited a case where a man was said to have died a drunkard because a certain doctor had prescribed brandy for him. It was subsequently learned that the man was said to have died a drunkard because a certain doctor had prescribed brandy for him. It was subsequently learned that the man was a drunkard before he ever saw the doctor.

Dr. H. C. Wood said that he was reminded that he had forgotten to refer to the use of alcohol as an antiseptic. In simple alcohol we have an antiseptic agent which is capable of banishing Listerism from surgery, if properly used. He would simply dress the stump with a cloth kept constantly wet with alcohol. No germs could possibly enter the wound, and the remedy is much safer than carbolic acid. He could not admit, however, that alcohol exerts this same antiseptic effect after entering the blood: the protoplasm of the blood cells is just as delicate as the protoplasm of the micrococci. Such treatment would be like the proposal to kill trichinæ in the muscles in giving the patient picric acid: before it poisons the parasite, the patient becomes translated.

He agreed with the last speaker in condemning the abuse of alcohol as an antipyretic. Alcohol should not be used in a routine manner in fevers, but should be held in reserve. It is of use in low fevers in other ways than as an antipyretic. From a number of calorimetric observations upon animals he had concluded that alcohol had no influence upon tissue-change or the production of animal heat, but does exert an influence upon the throwing out of heat from the blood. The temperature falls because more heat is dissipated, for the same reason that it falls after section of the spinal cord. Vaso-motor palsy leads to a decline in the temperature, whether caused by injury or by alcohol. It is very plain that if alcohol is to be given to produce the reduction of heat in this way it will have to be given in large doses—large enough to produce vaso-motor paralysis and depression, which we do not want caused in low fevers, and therefore cannot usually give it to reduce the heat to the normal temperature. As regards the quantity required he would not lay down any absolute rule. He refered to a case in which a man, after a rattlesnake-bite, took about two pints of whisky with only good result: an amount which in health would have depressed the heart, only elevated its power under these circumstances. It is so in low fevers. Eight or ten ounces per diem in a low fever may produce no more effect than two or three ounces in health. A young lady suffering with typhoid fever under his care had a pulse of 140. He increased the stimulant until she was taking one ounce of whisky every three hours; but finding it impossible to keep it down below this point, as it was at the rate of 160 at the time of his visit one night, he increased the stimulant to two ounces every two hours. The next morning it was down to 120. It seems, in some cases of low fever, that an amount of alcohol, which could produce fatal depression in health, may only act as a vital stimulant.

Dr. Addinell Hewson saw something inconsistent in the last speaker's remarks. He had spoken of the effects of alcohol in retarding cell-growth when referring to its antiseptic effect; he had also mentioned an effect upon the blood-cells and in retarding the movements of the white corpuscle. As all tissue change is dependent upon cell-growth, the speaker did not see how alcohol could influence the cell-movements without at the same time effecting tissue change. This is the teaching of experience; and it therefore happens that alcohol often impairs nutrition rather than benefits it. The effect of alcohol upon the gastric juice is to precipitate the pepsin. To these facts may be attributed the injurious effects of alcohol in some depressing diseases.

Dr. C. B. Nancrede said that the alcohol-dressing of wounds was an old treatment. He thought that experiments made many years ago by Onimus, of Montpellier, upon animals, demonstrated that it was not necessary to import micrococci in order to account for superation, showing, in fact, that the circumstances favoring suppuration and those developing micrococci are identical. He thought that there was danger of these experiments being overlooked. Fluids swarming with bacteria, which produced active effects upon the organizm, were treated with bromine, chromic acid, and acetic acid, which were found to destroy the power of the septic influence of the fluids, but not the number or activity of the vibrios.

Dr. Toboldt said that he had seen bacteria flourishing in a sixty-per cent. solution of alcohol, where they remained for over a year without obvious change.

Dr. Hamilton noticed the diversity of practice. Some physicians say that they cannot get along without alcohol in the treatment of typhus and typhoid fevers, and others, on the contrary never use it at all. If the same results always followed the alcohol treatment, its use would be more general.

Dr. J. T. Eskridge said that no precise rule could be laid down as regards the amount to be used. In one case he had given thirty-six ounces of brandy daily for several days, with successful result. It was in the fourth week of a relapse of typhoid fever. The patient did not take much alcohol in the first attack, but in the relapse the temperature reached 105°, the pulse 160. Brandy was increased to one and a half ounces every hour, and continued for about six days. The patient finally recovered. As the pulse came down to 110 and the vital foces were restored, the stimulant was gradually reduced.

The speaker also referred to the value of stimulants in atonic dyspepsia, given directly after eating.

Dr. O'Hara had not seen adduced any satisfactory evidence that alcohol is a food. It is not used to nourish young children, nor to build up the strength of prize-fighters when in training. He agreed with the lecturer as to its value in low fevers, where it does seem to supply force to the patient. He regarded the whole question of its food-value, however, as still *sub judice.*

Dr. Bartholow said that he had no doubt whatever that the position taken by the writers that alcohol is in a very limited sense a food is correct, and one supported by the authorities of our time. Of this there can be absolutely no doubt: a substance undergoing oxidation in the organism is in the position of other hydro-carbons in the food. Alcohol is very valuable as a food, for it spreads out over the whole surface of the lungs, where it yields its force very promptly, as it is very readily oxidized.

In regard to its antiseptic action, he could speak of its good effects in preventing local putrefaction. For eight years he had served as military surgeon, and had had charge of several large military hospitals: he therefore spoke of what he had seen when he says that it is entitled, in his opinion, to the place scarcely inferior to carbolic acid. In referring to what had been said in regard to the septic character of fluids independent of minute organisms, he said that it is true that there is a substance different from these bodies which can produce injurious effects; but the speaker had evidently not seen the report of Burdon Sanderson's experiments to the Privy Council, from which the conclusion to be drawn is that minute organisms play a very important part in the production of sepsis, but they are not septic themselves. It is not the minute organisms, but their action, that concerns us, while it is seen that their discussion is a very important subject, as it concerns the development of poisons connected with their presence.

Dr. W. R. D. Blackwood said that from an extended experience as an army surgeon and in private practice, he could endorse the alcohol treatment of wounds. He had not seen better results from Listerism. He had at the present time two stumps under treatment, which had had nothing on them but alcohol. He was perfectly satisfied with the dressing.

Dr. W. H. Parish said that brandy is a very valuable remedy in cholera infantum; cases are lost for not resorting to it sufficiently early. He referred to genuine cholera infantum, and not to entero-colitis. He had found that it would stop the vomiting; he also applies cloths wet with whisky to the epigastric region. Too much reliance is placed generally upon minute doses of calomel, and the brandy is left until it is too late. He agreed with Dr. Bartholow regarding the proper time of administering stimulants and the importance of proper dilution Hypodermic injections of brandy or whisky in surgical cases lessen shock and the danger of hemorrhage. They should not be given with a view of counteracting the effects of ether, which should never be pushed to its depressing effect in such cases.

Dr. W. S. Stewart noticed that the stimulants in low fevers do not intoxicate the patient, and cannot be detected in the breath even after large doses.

Dr. C. H. Thomas said that the question whether alcohol is or is not a food is an important one; but there can be no question but that in many cases it acts like a food. He cited the case of a lady who received a nervous shock and was almost insane in consequence. She was constantly vigilant, would take no food whatever, and was rapidly losing flesh: in ten days she lost between thirty and forty pounds. It was determined, on the third or fourth day, to give her whisky, of which she took some every hour; amounting to a quart a day. At the end of the tenth day she slept well, and for the first time complained of the amount of whisky, which was rapidly reduced—the next day to one pint, and the following day to half a pint. In a few days more after she had begun to take food regularly, she could not take a wineglassful of whisky without its flushing her face. In this case alcohol acted like a food, at least until other food could be taken.

Dr. Nancrede said that as a comparison had been made between the results of local use of alcohol and those of Lister's method, he would say that he was not an advocate of Listerism, but would enquire whether any surgeon in Philadelphia had adopted fully the genuine treatment as recommended by Lister. He thought not. We are therefore not in a position to speak of its results from experience.

Dr. Bartholow, in reply to a question, said that alcohol should not be given hypodermically to relieve the narcotic effect of ether or chloroform, because, having similar physiological effects and acting like them, it will only add fuel to the fire.

Dr. H. C. Wood, in conclusion, said that in regard to the question of alcohol acting as a food in childhood, it is paradoxical but true that if you wish to make a stunted child grow you should use alcohol, and if you wish to stunt a child you can do so by alcohol, simply because small amounts aid digestion, and large amounts disturb it and prevent assimilation. With regard to the non-appearance of alcohol in the breath in low fevers, he would account for it on the ground that alcohol was used up in the system.

